# Supplementary material for: Role of the two-component system AmgRS in early resistance of Pseudomonas aeruginosa to cinnamaldehyde
Source: Microbiol Spectr. 2024 Dec 10;13(1):e01699-24. doi: 10.1128/spectrum.01699-24 (PMC11705830; doi:10.1128/spectrum.01699-24)
Supplement: Supplemental material — Figures S1 and S2; Tables S1 to S3. [file spectrum.01699-24-s0001.docx]

**Supplementary data**

**B**

**A**

**C**

**Figure S1: Impact of CNA exposure on genes *oprM* (A), *nalC* (B), and *nalD* (C) expression.** The mRNA amounts in strains PA14, PA14∆*armR*, PA14∆*amgRS*, and PA14∆*amgRS*∆*amrR* (PA14∆∆) were determined at t_0_ (white bars), t_30 min_ (light grey), t_1h_ (dark grey) and t_2h_ (black) during exposure to 350 µg/mL CNA in exponentially growing bacteria (*A*_600 nm_ of 0.75). The amounts of specific cDNA were assessed on a Rotor Gene RG6000 instrument (Qiagen) by using the QuantiTect SYBR green PCR kit (Qiagen). The values from three biological replicates were averaged for each strain, normalized to that of housekeeping gene *rpsL,* and finally expressed as ratio to the transcript levels of untreated wild-type PA14 strain cultured in Mueller-Hinton broth supplemented with 0.3% DMSO, the solvent used for CNA dissolution. Anova test was performed on the data followed by a Dunnett test comparing each time to t0, and comparing each strain to PA14 for one selected time. Friedman test followed by Wilcoxon test were realised for PA14 and PA14Δ*armR* strain in the case of *nalC*. **p*_value<0.05; ***p*_value<0.01; ****p*_value<0.001

**Figure S2: Impact of CNA exposure on *mexY* expression.** The mRNA amounts in strains PA14, PA14∆*armR*, PA14∆*amgRS*, and PA14∆*amgRS*∆*amrR* (PA14∆∆) were determined at t_0_ (white bars), t_30 min_ (light grey), t_1h_ (dark grey) and t_2h_ (black) during exposure to 350 µg/mL CNA in exponentially growing bacteria (*A*_600 nm_ of 0.75). The amounts of specific cDNA were assessed on a Rotor Gene RG6000 instrument (Qiagen, Courtaboeuf, France) by using the QuantiTect SYBR green PCR kit (Qiagen). The values from three biological replicates were averaged for each strain, normalized to that of housekeeping gene *rpsL,* and finally expressed as ratio to the transcript levels of untreated wild-type PA14 strain cultured in Mueller-Hinton broth (MHB) supplemented with 0.3% DMSO, the solvent used for CNA dissolution. Gene *mexY* was considered overexpressed when its transcript level was ≥ 5-fold those of untreated wild-type strain PA14 (1). Anova test was performed on the data followed by a Dunnett test comparing each time to t0, and comparing PA14Δ*amgRS* to PA14 for one selected time. **p*_value<0.05; ***p*_value<0.01.

**Table S1: Transcriptomic data of AmgRS-regulated genes in response to CNA exposure (350 µg/mL)**

| **PA number** | **Gene** | **Function** | **Relative expression level compared with PA14 non-treated cells** | |
| --- | --- | --- | --- | --- |
|  |  |  | *t* = 15 min | *t* = 30 min |
| PA0425 | *mexA* | Adaptative protein of MexAB-OprM | **5.01** | **2.56** |
| PA0426 | *mexB* | RND Efflux pump of MexAB-OprM | **2.95** | 1.78 |
| PA2604 | *yccA* | Hypothetical protein | **4.75** | 1.57 |
| PA2830 | *htpX* | Protease | **5.32** | 1.05 |
| PA3712 | *yebE* | Hypothetical protein | **9.66** | **3.12** |
| PA5528 |  | Hypothetical protein | **4.13** | 1.10 |

*yegH, sugE, ygiT, yceJ, nlpD* and *mexXY* also belong to the AmgRS regulon (2), but were not significantly **(> 2-fold**) overexpressed at these exposure times.

**Table S2: primers used in PCR and RT-qPCR experiments**

| **Primers** | **Sequence (5’🡪 3’)** | **Source** |
| --- | --- | --- |
| ***Gene inactivation*** (complementary regions are indicated in red and green colours) | |  |
| ***amgRS*** |  |  |
| PA14*amgRS*-1 | CCCCCCCCTGCAGGTCGACGGCTTGCCCTGTTCCTCGATG | This study |
| PA14*amgRS*-2 | AGTATCAGGCCGGCAGGGTTCGACATAG |  |
| PA14*amgRS*-3 | AACCCTGCCGGCCTGATACTCGACGGGTTTG |  |
| PA14*amgRS*-4 | AGGCCTTAAGATCATATGCACACAAGCAACGCGTCGAAG |  |
| PA14*amgRS*-intF | CCTACAACGCCTGGTACACC |  |
| PA14*amgRS*-intR | GTTCGACCCAGGTGCCTTC |  |
| ***armR*** |  |  |
| iPA14*armR-1* | CTGTTTTGGCAAGCACTTCC | (3) |
| iPA14*armR-2* | TCTGATAGCGTCACCAGTTCATCCCCGTC |  |
| iPA14*armR-3* | ACTGGTGACGCTATCAGAATCCGCGCAGA |  |
| iPA14*armR-4* | GATTCCGCCTGCCATGAAG |  |
| ***RT-qPCR*** |  |  |
| ***mexB*** |  |  |
| *mexB*1 | ATCCGCCAGACCATCGCCA | (3) |
| *mexB*2 | CATCACCAGGAACACGAGGAGG |  |
| ***mexY*** |  |  |
| *mexY*1A | TTACCTCCTCCAGCGGC | (3) |
| *mexY*1B | GTGAGGCGCGCGTTGTG |  |
| ***nalC*** |  |  |
| *nalC*_Fw | GGCCTTTCTCGAACACGGTT | This study |
| *nalC*_Rv | GATCACCGCGGCAAACAG |  |
| ***nalD*** |  |  |
| *nalD*_Fw | AGATGCTCAACCAGGTACGC | (3) |
| *nalD*_Rv | GTTCCTCGGTGAATTCGCAAC |  |
| ***mexR*** |  |  |
| *mexR*_Fw | GAACTACCCCGTGAATCCCG | (3) |
| *mexR*_Rv | CGGACCAGGTTTCTTCCCTC |  |
| ***oprM*** |  |  |
| *oprM*_Fw | GCTCGCTGATCCCCGACTAC | This study |
| *oprM*_Rv | GTCGCGGTTGTTTTCCAGCG |  |
| **PA5528** |  |  |
| PA5528_Fw | CTGCTCGCCTCCATGTTCAT | This study |
| PA5528_Rv | CTGCCCCTGGTTCTGCTG |  |
| ***rpsL*** |  |  |
| *rpsL*_Fw | GCAACTATCAACCAGCTGGTG | (3) |
| *rpsL*_Rv | GCTGTGCTCTTGCAGGTTGTG |  |

**Table S3. Strains and plasmids used in this study**

| **Strains** | **Description** | **References** |  |
| --- | --- | --- | --- |
| ***Pseudomonas aeruginosa*** | | | |
| PA14 | Wild-type reference strain, susceptible to antibiotics | (4) |  |
| PA14∆*mexAB* | *mexAB* deletion mutant from PA14 | (3) |  |
| PA14∆*amgRS* | *amgRS* deletion mutant from PA14 | This study |  |
| PA14∆*armR* | *armR* deletion mutant from PA14 | (3) |  |
| PA14∆*armR*∆*amgRS* | *armR* and *amgRS* deletion mutant from PA14 | This study |  |
| PA14-*lux* | Strain PA14 rendered bioluminescent by chromosomal integration of plasmid pUC18T-MiniTn*7*-P1-*lux* | (3) |  |
| PA14∆*mexAB*-*lux* | Mutant PA14∆*mexAB* rendered bioluminescent by chromosomal integration of plasmid pUC18T-MiniTn*7*-P1-*lux* | (3) |  |
| PA14∆*amgRS*-*lux* | Mutant PA14∆*amgRS* rendered bioluminescent by chromosomal integration of plasmid pUC18T-MiniTn*7*-P1-*lux* | This study |  |
| PA14∆*armR*∆*amgRS-lux* | Mutant PA14∆*armR*∆*amgRS* rendered bioluminescent by chromosomal integration of plasmid pUC18T-MiniTn*7*-P1-*lux* | This study | |
| ***Escherichia coli :*** | | | |
| DH5α | *F- φ80lacZΔM15 Δ(lacZYA-argF) U169 recA1 endA1 hsdR17(rk. mk+) phoA supE44 thi-1 gyrA96 relA1 λ*^-^ | Invitrogen |  |
| CC118*λpir* | CC118 lysogenic for phage *λpir* | (5) |  |
| HB101 | *subE44 subF58 hsdS3*(r_B_^-^ m_B_^-^) *recA13 ara-14 proA2 lacY1 galK2 rpsL20 xyl-5 mtl-1* | (6) |  |
| DH5α(pTNS3) | Strain DH5α containing helper plasmid pTNS3 for chromosomal insertions (encoding the Tn*7* site-specific transposition pathway; Amp^R^) | (7) |  |
| HB101(pRK2013) | Strain HB101 containing mobilising plasmid pRK2013 for conjugation (*mob1. tra1. colE1*; Kan^R^) | (8) |  |
| CC118*λpir*(pKNG101) | Strain CC118*λpir* containing suicide vector pKNG101 (*oriR6K. sacB*; Str^R^) | (9) |  |
| XL1-Blue(pUC18T-MiniTn7-P1-*lux)* | Strain XL1-Blue containing self-proficient integration vector pUC18T-MiniTn*7*-*luxCDABE* (viral promoter P1; Amp^R^. Gen^R^) | (10) |  |

Amp^R^, Kan^R^, Str^R^, Gen^R^: selective plasmid markers conferring resistance to ampicillin, kanamycin, streptomycin, and gentamicin, respectively.

**References**

1. Llanes C, Pourcel C, Richardot C, Plésiat P, Fichant G, Cavallo JD, Merens A, Group GS. 2013. Diversity of beta-lactam resistance mechanisms in cystic fibrosis isolates of *Pseudomonas aeruginosa*: a French multicentre study. J Antimicrob Chemother 68:1763-71.

2. Lee S, Hinz A, Bauerle E, Angermeyer A, Juhaszova K, Kaneko Y, Singh PK, Manoil C. 2009. Targeting a bacterial stress response to enhance antibiotic action. Proc Natl Acad Sci U S A 106:14570-5.

3. Tetard A, Zedet A, Girard C, Plésiat P, Llanes C. 2019. Cinnamaldehyde induces expression of efflux pumps and multidrug resistance in *Pseudomonas aeruginosa*. Antimicrob Agents Chemother 63.

4. Liberati NT, Urbach JM, Miyata S, Lee DG, Drenkard E, Wu G, Villanueva J, Wei T, Ausubel FM. 2006. An ordered, nonredundant library of *Pseudomonas aeruginosa* strain PA14 transposon insertion mutants. Proc Natl Acad Sci U S A 103:2833-8.

5. Herrero M, de Lorenzo V, Timmis KN. 1990. Transposon vectors containing non-antibiotic resistance selection markers for cloning and stable chromosomal insertion of foreign genes in Gram-negative bacteria. J Bacteriol 172:6557-67.

6. Lacks S, Greenberg B. 1977. Complementary specificity of restriction endonucleases of *Diplococcus pneumoniae* with respect to DNA methylation. J Mol Biol 114:153-68.

7. Choi KH, Mima T, Casart Y, Rholl D, Kumar A, Beacham IR, Schweizer HP. 2008. Genetic tools for select-agent-compliant manipulation of *Burkholderia pseudomallei*. Appl Environ Microbiol 74:1064-75.

8. Ditta G, Stanfield S, Corbin D, Helinski DR. 1980. Broad host range DNA cloning system for gram-negative bacteria: construction of a gene bank of *Rhizobium meliloti*. Proc Natl Acad Sci U S A 77:7347-51.

9. Kaniga K, Delor I, Cornelis GR. 1991. A wide-host-range suicide vector for improving reverse genetics in Gram-negative bacteria: inactivation of the *blaA* gene of *Yersinia enterocolitica*. Gene 109:137-41.

10. Damron FH, McKenney ES, Barbier M, Liechti GW, Schweizer HP, Goldberg JB. 2013. Construction of mobilizable mini-Tn7 vectors for bioluminescent detection of Gram-negative bacteria and single-copy promoter *lux* reporter analysis. Appl Environ Microbiol 79:4149-53.
